# Prenatally diagnosed rare giant congenital epulis: a case report

**DOI:** 10.1515/crpm-2026-0001

**Published:** 2026-05-25

**Authors:** Greta Miškinaitė, Gabija Asipauskaitė, Gabija Gaidamavičienė, Eglė Machtejevienė, Valdas Šarauskas, Ilona Aldakauskienė

**Affiliations:** Medical Academy, Faculty of Medicine, Lithuanian University of Health Sciences, Kaunas, Lithuania; Department of Neonatology, Hospital of Lithuanian University of Health Sciences, Kaunas, Lithuania; Department of Obstetrics and Gynecology, Hospital of Lithuanian University of Health Sciences, Kaunas, Lithuania; Department of Pathological Anatomy, Hospital of Lithuanian University of Health Sciences, Kaunas, Lithuania

**Keywords:** neonatal, oral lesions, congenital epulis, prenatal diagnostics

## Abstract

**Objectives:**

Congenital epulis (CE) is a rare benign tumour typically arising from the alveolar ridge and more frequently observed in female neonates. The report highlights its potential impact on neonatal breathing and feeding, emphasizing the importance of early recognition and management.

**Case presentation:**

We present a case of a female neonate with a giant CE detected on prenatal ultrasound. After the birth the lesion interfered with feeding, while respiratory function remained unaffected. Three masses were surgically dissected from the maxillary mucosa and the neonate successfully transitioned to oral feeding within the first week. Histopathological examination revealed a granular cell tumour with negative immunoreactivity for S100.

**Conclusions:**

The case underlines the significance of prenatal diagnosis, collaborative multidisciplinary planning, and, when indicated, the application of the EXIT procedure for congenital orofacial tumours with potential airway obstruction. Early recognition and integrated care can substantially improve neonatal prognosis.

## Introduction

Congenital epulis (CE) is a sporadic benign mesenchymal tumour arising from the maxillary alveolar ridge, followed by the mandibular alveolar ridge at a ratio of 3:1 [1], 2]. It most commonly presents as an exophytic mass protruding from a newborn’s mouth and typically appears as a smooth-surfaced lesion, either sessile or a pedunculated, usually red in colour [2]. In most cases, it is evident at birth or in early infancy, whereas prenatal diagnosis is achieved in only a few cases [3], 4]. The histogenesis of the lesion remains unclear and controversial, while the lesion itself is extremely rare, occurring in approximately 0.0006 % cases [1], 5]. To explain the origin, several hypotheses have been proposed, including pericytic, fibroblastic, histiocytic, neural and undifferentiated mesenchymal lineages [5]. A potential hormonal role is also suggested by the fact that this lesion is 10 times more common in female newborns than in males [6].

Large tumours can restrict the airways and oral cavity during pregnancy and the neonatal period, posing a risk of airway obstruction and interfering with the feeding and/or mouth closure, which usually necessitates surgical intervention [3], 7]. For this reason, prenatal diagnosis of CE plays a crucial role in ensuring optimal perinatal management and surgical preparation. We report a case of a neonate with giant CE prenatally detected on the upper palate.

## Case presentation

**Figure 1:**
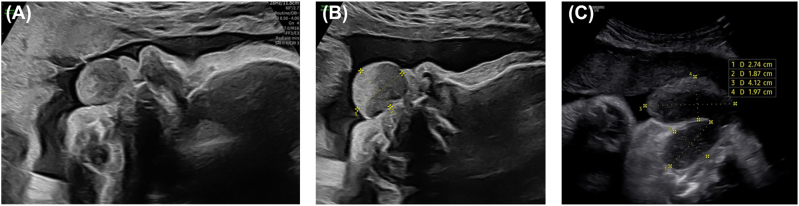
Prenatal ultrasound images: (A) masses (epulis) that surge through the fetus’s mouth at 32 weeks and 4 days of gestation; (B) progressively enlarging epulis at 33 weeks and 6 days of gestation; (C) two components of the epulis at 37 weeks and 0 days of gestation.

A 28-year-old primigravida was referred to the Fetal Medicine Centre due to a suspected fetal facial abnormality at 32 weeks and 4 days of gestation. Prenatal ultrasound confirmed normal fetal growth but revealed a soft tissue mass measuring 27 × 19 mm arising from the upper lip or alveolar ridge (Figure 1A). Fetal swallowing was observed through both the mouth and nose, and tongue mobility was unimpaired.

**Figure 2:**
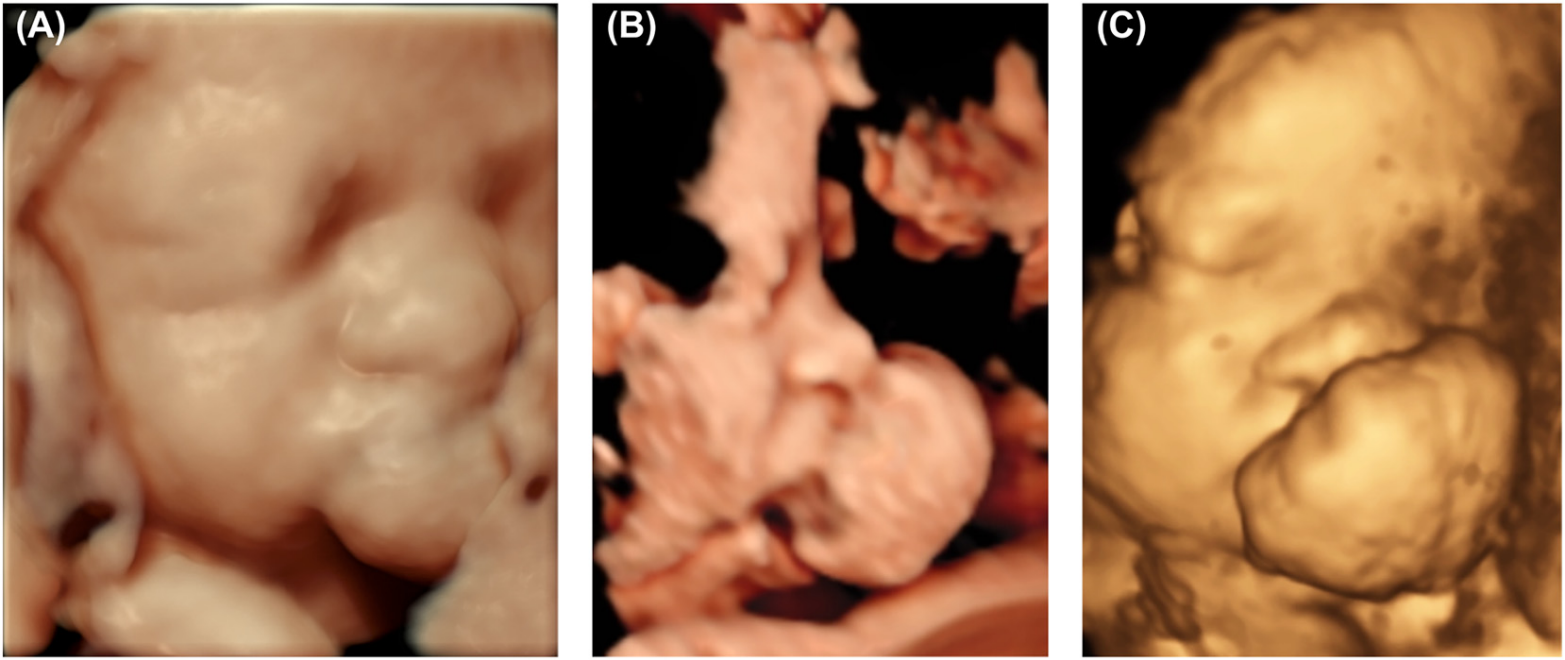
3D ultrasound images of epulis: (A) 33 weeks and 6 days of gestation, (B, C) giant epulis at 37 weeks and 0 days of gestation.

At 33 weeks and 6 days, a multidisciplinary case conference was held, involving obstetricians, neonatologists, paediatric surgeons, otolaryngologists, and craniofacial surgeons. The origin of the mass remained unclear, with a slight increase in size to 29 × 19 mm (Figures 1B and 2A). Given the potential for airway obstruction at birth, delivery in a tertiary perinatology centre with a team prepared for advanced airway management, including possible intubation, was recommended.

**Figure 3:**
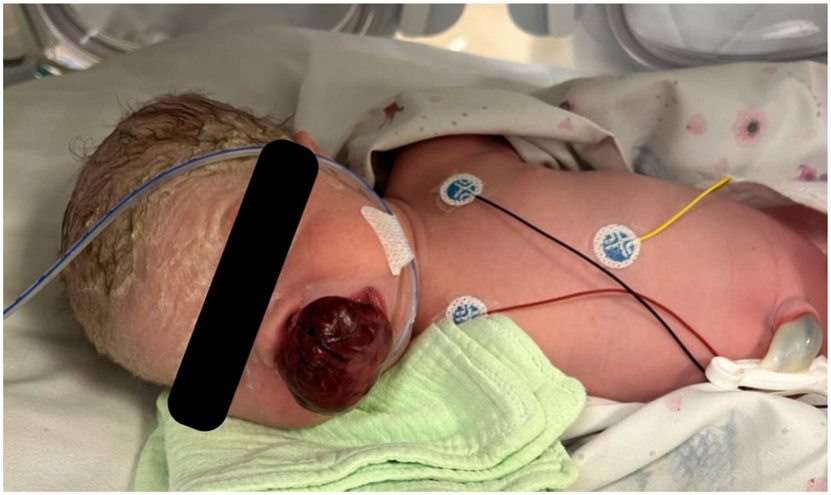
The neonate with a giant epulis measuring an extraoral component of 37 mm and an intraoral component of 22 mm in diameter.

At 37 weeks of gestation, prenatal ultrasound demonstrated two separate soft tissue masses in the region of the upper palate, measuring 27 × 18 mm and 41 × 19 mm, respectively, indicating further progression. The patient presented with spontaneous onset of labour. After reevaluation and multidisciplinary consultation, the decision was made to perform a caesarean section with an EXIT procedure. A female neonate was delivered weighing 2,786 g, with Apgar scores of 8 and 9 at 1 and 5 min, respectively (Figure 3).

The neonate had adequate spontaneous respiratory effort immediately after birth. Continuous positive airway pressure (nCPAP) was initiated to provide respiratory support, and umbilical cord was clamped at 2 min of life. Following stabilization, the infant was transferred to the neonatal intensive care unit (NICU), where nCPAP was subsequently discontinued. Respiratory function remained stable with no requirement for supplemental oxygen. Cardiovascular condition was stable. Due to impaired oral feeding secondary to the lesion, a gastric tube was placed, and enteral nutrition was initiated, which was well tolerated.

During the first day, one of the masses spontaneously detached and was expelled from the mouth. Histopathological examination revealed that the specimen consisted of necrotic tissue. Complete blood count, C-reactive protein, arterial blood gas, electrolytes, blood glucose and bilirubin levels were within normal ranges.

**Figure 4:**
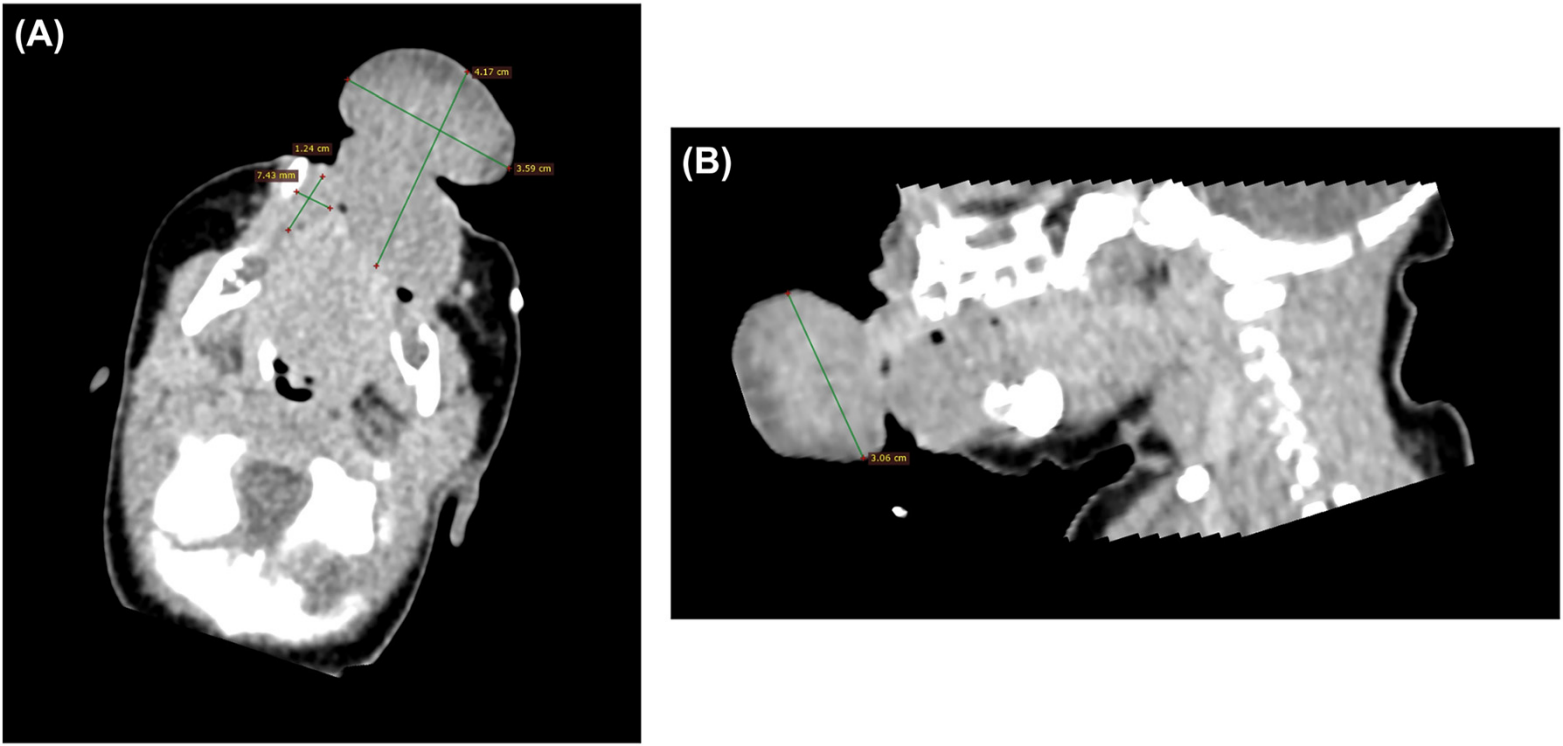
CT images of the lesion: (A) Axial CT image showing the lesion, total length 41 mm, extraoral component 35 mm in diameter, with a smaller similar lesion measuring 12 × 7 mm. (B) Sagittal CT image demonstrating the lesion, vertical extent measured 30 mm.

On the 3rd day of life, a multidisciplinary team meeting decided to perform computed tomography (CT) to assess the size, location and involvement with surrounding structures. Due to the potential risk of airway complications associated with the lesion, a CT scan was performed during natural sleep. The images revealed a well-defined, solid soft-tissue mass with an hourglass configuration involving both intraoral and extraoral regions on the left side of the oral cavity. The lesion measured approximately 41 mm in length and 30 mm in vertical dimension, with an extraoral component of about 35 mm and an intraoral component of approximately 22 mm in diameter. The intraoral portion extended widely, coming into contact with the mucosal surfaces of the left cheek, lips, and both upper and lower vestibular folds (more prominently inferiorly) as well as the gingiva of both jaws. Due to the extensive mucosal contact, the exact site of attachment could not be determined. Additionally, a smaller lesion of similar density and enhancement pattern (measuring 12 × 7 mm coronally) was suspected along the right mandibular buccal gingiva. The tongue was located dorsally to the described masses and demonstrated homogeneous contrast enhancement. The lumina of the pharynx, larynx, and upper trachea were patent and without evidence of deformation (Figure 4).

The mass remained non-haemorrhagic, was routinely irrigated with 0.9 % saline solution and kept moisturized with sterile oil until surgery. On the fourth day of life, acute haemorrhage from the superior portion of the mass was observed. Haemostatic sponge was applied and haemostasis was achieved. Throughout the episode, vital parameters remained stable, and the patient’s airway remained patent.

**Figure 5:**
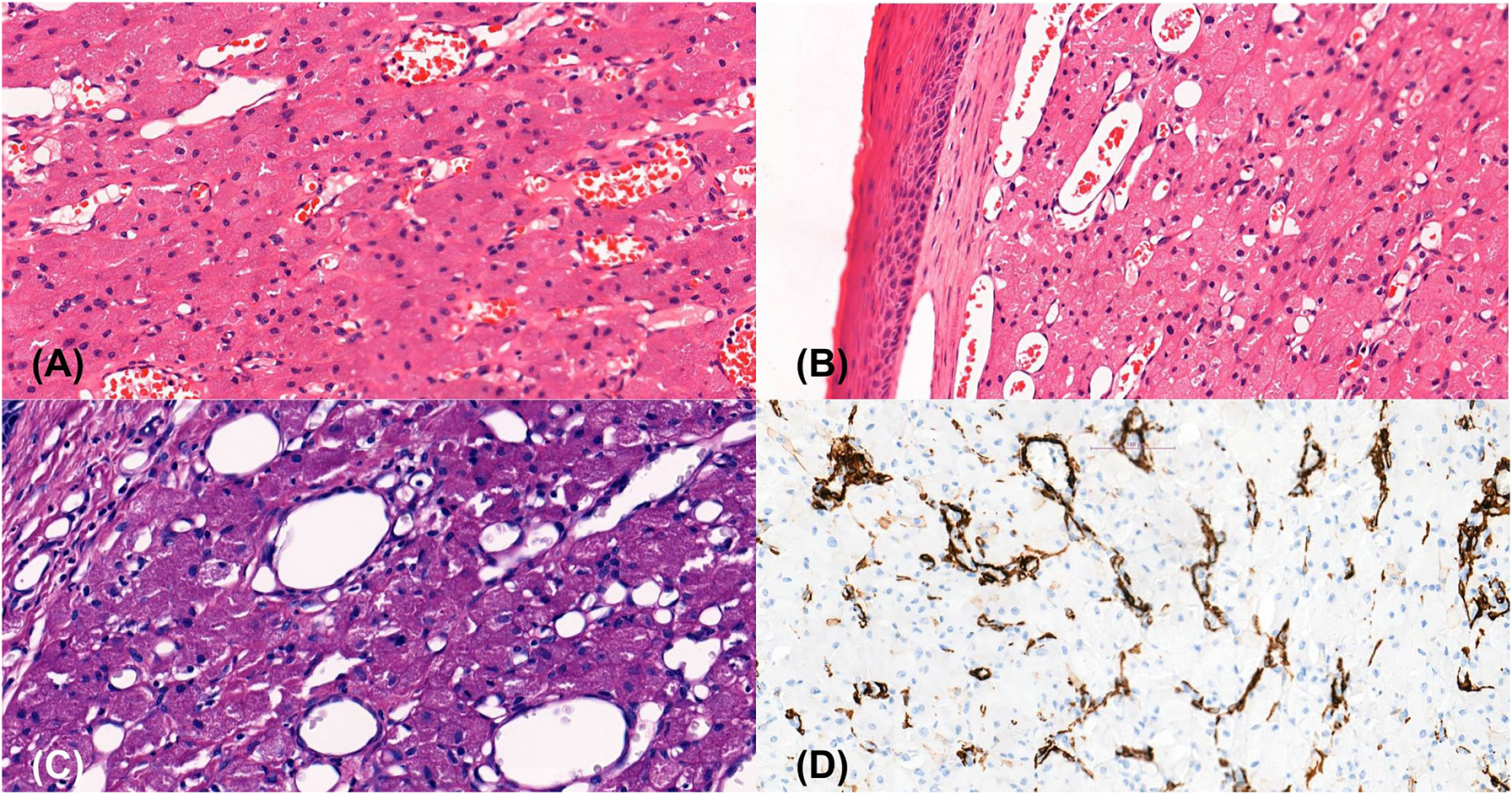
Histological images of the lesion: (A) and (B) haematoxylin and eosin. (C) PAS-positive cytoplasmic granules. (D) Smooth-muscle actin (SMA).

On the 4th day of life radical excision of the oral cavity masses was performed using monopolar electrocautery under general anaesthesia with endotracheal intubation. Three distinct masses were separated from the maxillary mucosa and submitted for histopathological evaluation. Histopathological examination of the masses obtained during surgery revealed that the examined tissue fragments consist of a cellular neoplastic tissue formed by trabecular or solid structures composed of mitotically inactive tumour cells with small nuclei and abundant granular oxyphilic cytoplasm containing PAS-positive, diastase-resistant granules. The tumour cells show negative immunoreactivity for actin, desmin, SO×10, and S100 (Figure 5).

The surgical procedure was completed without any complications. The patient was transferred back to the NICU while still intubated. Extubation was performed 3 h postoperatively, after which spontaneous respiration was adequate with no signs of respiratory distress. No episodes of haemorrhage were observed during the postoperative follow-up period, and the overall condition remained stable. Breastfeeding was successfully initiated on the sixth day of life.

A mucosal lesion resembling a fibroma, measuring up to 5 mm, persisted on the dorsal surface of the tongue. The lesion was left under further observation, and urgent re-evaluation was advised if it shows any growth.

The patient remained clinically stable throughout the postoperative period. The operative site was healing without bleeding. Spontaneous respiration was regular and unlaboured. The neonate was breastfed on demand and had an appropriate weight gain. Consequently, on the 10th day of life, the patient was discharged home.

## Discussion

CE is an uncommon benign lesion that develops on the alveolar ridge of the jaw in newborns [8]. When detected prenatally, the lesion may continue to grow over the following weeks; however, its growth typically ceases abruptly after birth [9]. There are no known hereditary predispositions, and the lesions appear sporadically. While CE is generally solitary [10], multiple occurrences have been documented in about 10 % of cases [11]. The most common site is the upper maxillary, but it can also occur in the mandibular region and, in rare instances, the tongue [12]. In the presented case, three lesions were dissected from the maxillary mucosa. A fibroma-like mass was noted on the ventral surface of the tongue apex measuring up to 5 mm. This lingual lesion was left for further observation. Additional histopathological studies would be necessary to determine whether it might represent another focus of CE but such investigations have not been performed yet.

### Differential diagnosis

When evaluating congenital masses, other possible oral lesions should always be considered to ensure an accurate diagnosis. Although congenital oral masses have a wide range of differential diagnosis, determining their anatomical position and consistency is essential for clarifying the diagnosis. The anatomical site of the lesion is particularly helpful in narrowing the differential diagnosis. For instance, midline palatal masses are often associated with epignathus, teratoma, malignant tumours or masses, lesions involving the maxilla or mandible are commonly epulis. Multiple neck spaces are mostly associated with vascular abnormalities, malignant mass. While those located on the tongue or the base of the mouth may represent oral foregut cyst, ranula, choristomas. Moreover, congenital oral masses in newborns can be broadly divided into cystic and solid lesions. Cystic lesions include gingival cysts of the newborn, Bohn’s nodules, eruption cysts, dermoid cysts, mucoceles, and ranulas, Rega-Fede disease and midpalatal raphe cysts of the newborn. Whereas solid lesions comprise haemangiomas, lymphangiomas, Langerhans cell histiocytosis, melanotic neuroectodermal tumour of infancy, teratomas, oral choristomas, malignant tumours and CE [2].

Beyond its anatomical and consistency–related characteristics, the lesion’s histological and immunohistochemical features play a critical role in its diagnostics. Histologically, CE consists of granular cells, which is why it is also commonly referred to as congenital granular cell epulis (CGCE). These granular cells resemble those found in Schwannian-derived granular cell tumours (GCT), and both are positive for vimentin, which explains their close affinity [9]. Granular cell tumour is typically derived from Schwann cells and exhibits a positive S100 reaction, whereas CE does not. In addition, CE as well does not show reactivity to NGFR/p75 and inhibin-alpha, although both CE and granular cell tumour are positive for macrophage markers like CD68 and Ki-M1P. In our presented case, histopathological examination revealed a granular cell tumour with negative immunoreactivity for S100, which supports the diagnosis of epulis. Despite variability in other immunohistochemical markers, the absence of S-100 reactivity remains the definitive diagnostic feature distinguishing CE from GCT [5], 9], 11]. Accurate diagnosis in complex cases can be facilitated through the integration of clinical features with microscopic and immunohistochemical findings.

Recent case-based literature further supports the distinction between congenital epulis and other neonatal oral masses. Pensabene et al. reported a full-term neonate with a 2 × 2 cm exophytic hemangioma of the hard palate causing feeding impairment and anemia. By contrast, CE typically appears at birth as a firm, polypoid mass on the maxillary alveolar ridge, halts further growth at delivery, and often regresses. Infantile hemangiomas usually become apparent in the first weeks of life and then proliferate rapidly, whereas epulis is already fully present at birth [13]. Similarly, neonatal alveolar lymphangioma presents as a translucent bluish cystic nodule that involutes within months [14]. Emphasizing these differences in clinical appearance, anatomical site, and course helps narrow the differential diagnosis. Immunohistochemical findings further support this distinction, as hemangiomas are typically GLUT1 positive, whereas congenital epulis is S100 negative. Together, these features confirm that the granular cell gingival tumor in our case is consistent with congenital epulis rather than a vascular or other neonatal mass [13].

### Prenatal and postnatal diagnostics

Although CE is often identified at birth, 3D ultrasound and magnetic resonance imaging (MRI) tests may identify it in pregnancy if the lesion is substantial [7], 15]. Anomaly scans conducted between 12 and 20 weeks of pregnancy are unlikely to diagnose CE because growth typically begins around the 22nd week [2]. However, CE can be diagnosed during the middle to late period of gestation by prenatal ultrasound [16]. Because large lesions may impede a normal vaginal delivery and necessitate a caesarean section, prenatal diagnosis is important when selecting the delivery technique [11]. Moreover, prenatal ultrasound examination plays a crucial role in enabling early communication with parents and reducing parental anxiety [5]. In the presented case, the tumour was identified during prenatal ultrasound. Caesarean section with EXIT procedure was scheduled after multidisciplinary team meeting.

For postnatal diagnosis of CE, MRI is often preferred over CT, as it better provides a more precise assessment of the extent and characteristics of soft tissue masses in newborns, while also eliminating the risk of ionizing radiation exposure [17]. Although MRI is widely considered as safe in neonates and is routinely performed under general anaesthesia in clinically stable infants, the requirement for anaesthesia remains a relevant factor in specific clinical contexts. In our case general anaesthesia posed significant risks due to airway-related risks, including laryngeal injury during laryngeal mask insertion, potential airway bleeding, air leakage, and ineffective ventilation. Sedation without airway intervention was also considered unsafe due to the risk of respiratory depression or arrest and the potential for difficult airway management in such case.

At the same time, CT imaging involves ionizing radiation, which is a particular concern in neonates given their higher susceptibility and the potential for long-term cumulative effects. For this reason, current paediatric imaging approaches place strong emphasis on reducing radiation exposure and favouring non-ionizing techniques whenever possible [18]. However, considering the airway-related clinical circumstances in this case, CT imaging performed during natural sleep was considered the most appropriate and safest diagnostic option.

### Tumour size and clinical significance

Although CE can range from 3 to 80 mm, the mass’s diameter is typically only about 10 mm [19]. Compared with most cases reported in the literature, our patient’s lesion was relatively large, with an extraoral component of 37 mm and an intraoral component of 22 mm in diameter. Large tumours can restrict the airways and mouth during pregnancy and the neonatal period, making the prenatal diagnosis of CE crucial [7]. In the reported case, the diagnosis was made prenatally, allowing precise planning of the delivery mode, scheduling of the procedure, and involvement of a multidisciplinary team. These measures ensured a safe delivery, as the team was prepared for potential airway emergencies. Fortunately, the tumour did not interfere with breathing, although it led to feeding difficulties.

### Surgical management

Tumour placement and size might result in mechanical obstruction, which can cause feeding difficulties, cyanosis, dyspnoea, and possibly even mortality from hypoxia during the perinatal and postnatal period[20]. Usually CE is surgically removed under local or general anaesthesia [12], there is no need for extensive surgical resection [21]. Spontaneous regression of CE has been also reported and may be considered in neonates with small lesions (<2 cm) that do not compromise the airway or feeding, particularly when families are compliant, making non-surgical management a viable option [15]. The treatment selected for our situation was radical excision of the oral cavity masses under general anaesthesia with endotracheal intubation. The aim was to remove the masses that interfered with feeding and posed risk of bleeding and airway obstruction.

### Prognosis

The long-term prognosis for patients with epulis is favourable. Small lesions may regress spontaneously, whereas larger ones require surgical excision. Recurrence of epulis is very rare [16], and there is no tendency toward malignancy [11]. Prenatal diagnosis enables multidisciplinary planning for a safe delivery, while postnatal management aims to secure airway patency and adequate feeding.

## Conclusions

This case highlights the importance of prenatal diagnosis, multidisciplinary planning, and the use of the EXIT procedure in managing congenital orofacial tumours with potential airway compromise. In these cases, CE management should be individualized and involve close collaboration among obstetricians, neonatologists, paediatric surgeons, radiologists, and anaesthesiologists. Early identification and coordinated care can significantly improve neonatal outcomes in such complex cases.
